# The DE and FG loops of the HPV major capsid protein contribute to the epitopes of vaccine-induced cross-neutralising antibodies

**DOI:** 10.1038/srep39730

**Published:** 2016-12-22

**Authors:** Sara L. Bissett, Anna Godi, Simon Beddows

**Affiliations:** 1 Virus Reference Department, Public Health England, London, UK

## Abstract

The human papillomavirus (HPV) vaccines consist of major capsid protein (L1) virus-like particles (VLP) and are highly efficacious against the development of cervical cancer precursors attributable to oncogenic genotypes, HPV16 and HPV18. A degree of vaccine-induced cross-protection has also been demonstrated against genetically-related genotypes in the Alpha-7 (HPV18-like) and Alpha-9 (HPV16-like) species groups which is coincident with the detection of L1 cross-neutralising antibodies. In this study the L1 domains recognised by inter-genotype cross-neutralising antibodies were delineated. L1 crystallographic homology models predicted a degree of structural diversity between the L1 loops of HPV16 and the non-vaccine Alpha-9 genotypes. These structural predictions informed the design of chimeric pseudovirions with inter-genotype loop swaps which demonstrated that the L1 domains recognised by inter-genotype cross-neutralising antibodies comprise residues within the DE loop and the late region of the FG loop. These data contribute to our understanding of the L1 domains recognised by vaccine-induced cross-neutralising antibodies. Such specificities may play a critical role in vaccine-induced cross-protection.

Oncogenic human papillomavirus (HPV) genotypes are the causative infectious agents of approximately 5% of all human cancers worldwide^1^. Infection with an oncogenic HPV genotype plays a fundamental role in the development of cervical carcinoma, one of the leading causes of cancer death in women and it is also an identified risk factor associated with the development of other anogenital cancers and head and neck cancers^2^. The oncogenic genotypes HPV16 and HPV18 account for *ca.* 70% of cervical cancer cases worldwide^3^ and the majority of the oncogenic HPV genotypes are closely related to either HPV16 or HPV18 within the Alpha-9 or Alpha-7 species groups, respectively.

The HPV double-stranded DNA genome is encapsidated within a non-enveloped, icosahedral structure comprised of the major (L1) and minor (L2) viral structural proteins^4^,^5^. The viral capsid is formed in a stepwise manner whereby five L1 monomers form an intermediate pentameric capsomer structure then 72 capsomers associate to form the icosahedral structure^6^. The occupancy of the L2 within the capsid is unclear but upper estimates of one L2 monomer per capsomer have been proposed^7^. Each L1 monomer consists of a core of β-strands and α-helix structures which support the five surface exposed loop regions designated BC, DE, EF, FG and HI^8^. The L1 protein mediates primary viral attachment via interactions between FG and HI loop lysine residues and host heparin sulphate moieties^9^.

Inter-genotype L1 amino acid sequence variation is mostly concentrated within the surface exposed loop regions^8^,^10^,^11^ and appears to dictate the predominantly type-specific nature of the L1 neutralising antibody response. HPV natural infection antibodies and the majority of type-specific monoclonal antibodies (MAbs) which neutralise HPV infectivity recognise one or more of these surface exposed loops^12^,^13^. Cryo-electron microscope analysis recently demonstrated that the epitope footprints recognised by a number of HPV16 MAbs include amino acid residues from multiple L1 loops^14^,^15^. The epitope of one of these MAbs, H16.V5, included loops from two neighbouring L1 monomers with the majority of contact residues predicted to be in the DE and FG loops with a minor number of contact residues located in the EF and HI loops^15^. In comparison, the epitope footprints recognised by four HPV31 MAbs appear to be restricted to amino acid residues within the FG loop^16^.

L1 virus-like particles (VLP) precipitated on an aluminium salt adjuvant are the basis of the prophylactic vaccines, Cervarix^®^ and Gardasil^®^, additionally Cervarix^®^ also contains monophosphoryl lipid A. Clinical trials have demonstrated that both vaccines are highly efficacious against the development of cervical cancer precursors and other anogenital diseases attributable to vaccine genotypes HPV16 and HPV18^17^. A degree of cross-protection has also been reported against oncogenic genotypes HPV31 and HPV33 which are related to HPV16 within the Alpha-9 species group and HPV45 which is related to HPV18 within the Alpha-7 species group^18^,^19^. Reductions in infections due to the vaccine types and HPV31, HPV33 and HPV45 have been reported in vaccinated populations^20^, supporting the findings of the vaccine efficacy trials. A third L1 VLP-based prophylactic vaccine, Gardasil^®^9, has recently been licensed for use following successful clinical trials^21^.

Vaccine type L1 neutralising antibodies can be detected in both the serum and cervicovaginal secretions of vaccine recipients^22^,^23^ and are assumed to mediate vaccine-induced type-specific protection, based upon preclinical passive transfer experiments^24^,^25^. These antibody specificities appear to recognise regions on the L1 surface exposed loops, for example HPV16 vaccine-induced neutralising antibodies can compete with and block the binding of the H16.V5 MAb to its L1 loop epitope^26^. L1 cross-neutralising antibodies can also be detected in the serum and cervicovaginal secretions of vaccine recipients^23^,^27^, although the role of such antibody specificities in mediating cross-protection is unclear.

Pseudovirions (PsV) are used as surrogates for authentic virions for the measurement of antibody-mediated neutralisation in a range of viral systems including HIV, influenza virus, SARS coronavirus and HPV^28^,^29^,^30^,^31^. HPV L1L2 PsV generally represent the genotype reference sequence^32^ and vaccine-induced neutralising antibodies recognise and bind L1 antigenic domains on these representative PsV^22^,^27^,^33^. Cross-neutralising antibodies are detected less frequently and at lower titres than vaccine type neutralising antibodies^22^,^27^,^34^. In addition, these cross-reactive antibody specificities demonstrate an essentially species-group specific reactivity with the breadth of the cross-neutralising antibody response differing between the Alpha-7 and Alpha-9 species groups^23^,^33^,^35^.

That cross-neutralising antibodies are generated in response to the L1 VLP-based HPV vaccines indicates that the L1 proteins of the vaccine genotypes harbour immunogenic domains that share sequence and/or structural similarity with the L1 proteins of non-vaccine genotypes. Within this present study L1 crystallographic homology models were used to predict structural differences in the loops between HPV16 and the non-vaccine Alpha-9 genotypes. These data informed the design and generation of chimeric PsV with inter-genotype loop swaps for the delineation of specific L1 domains recognised by HPV vaccine-induced cross-neutralising antibodies. The identification of such L1 antigenic domains will contribute to our understanding of virus-host interactions induced in response to vaccination.

## Results

### Generation of L1 homology models representing the Alpha-9 genotypes

The crystal structure of the HPV16 L1 capsomer (Protein Data Bank [PDB] Code: 2R5H) was used as the template to which the L1 amino acid sequences representing the Alpha-9 PsV were modelled. Three amino acid residues differ between the L1 sequences of the HPV16 crystal and PsV: two positons in the EF loop (Q177N and Q181N) and one position in the FG loop (A266T). These sequence differences did not appear to adversely impact upon the quality of the HPV16 L1 homology model which had a Global Model Quality Estimation (GMQE) score of 1.00. The L1 homology models representing the remaining Alpha-9 genotypes produced GMQE scores of 0.99 (HPV31 and HPV35) and 0.98 (HPV33, HPV52 and HPV58).

The crystal structure of the HPV35 L1 capsomer (PDB Code: 2R5J) was used as a control to evaluate the structural accuracy of the HPV35 PsV L1 homology model based upon the HPV16 crystal structure. The pairwise comparison of the homology model on the HPV35 crystal structure by superimposition demonstrated a root mean square (RMS) deviation of 0.68 Å.

### Prediction of L1 loop structural differences

**Figure 1:**
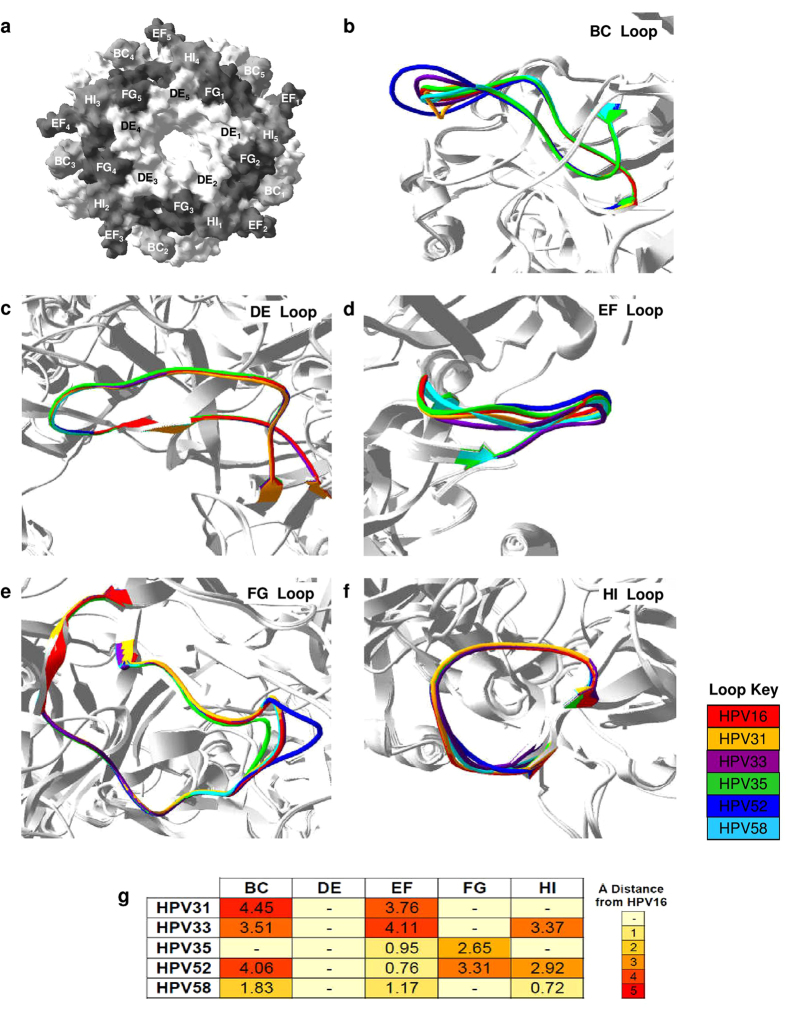
Predicted differences in L1 loop structure between homology models representing HPV16 and non-vaccine Alpha-9 genotypes. (**a**) Top view of HPV16 L1 surface-filled model with the BC, DE, EF, FG and HI loops highlighted. Expanded view of predicted structural differences in the BC (**b**), DE (**c**), EF (**d**), FG (**e**) and HI (**f**) with loops colour-coded by genotype. (**g**) Heatmap represents the predicted distance (mean Å) between the L1 loops of the homology models representing HPV16 and the non-vaccine Alpha-9 genotypes. Key indicates Å distance from HPV16 with (-) indicating that it was not possible to resolve any potential differences between loops.

Pairwise model comparisons were carried out between the HPV16 PsV L1 homology model and the L1 homology models of each individual non-vaccine Alpha-9 PsV. The L1 loops (Fig. 1a) were analysed for any predicted changes between the two models (Fig. 1b**–**f) with the distance between altered loop structures summarised (Fig. 1g). The superimposition of the non-vaccine Alpha-9 homology models onto the HPV16 homology model generated the following RMS deviations: HPV31 0.26 Å; HPV33 0.47 Å; HPV35 0.47 Å; HPV52 0.49 Å and HPV58 0.41 Å. The estimated loop distance between homology models was considered significant if the distance measured between HPV16 and a non-vaccine genotype for an individual loop was greater than the RMS value generated by the pairwise comparison of the homology models.

Structural changes in the BC loop were predicted for all non-vaccine genotypes except HPV35 (Fig. 1b), with the shifts in the BC loop compared to HPV16 ranging from a mean 1.83 ± (s.d.) 0.83 Å for HPV58 to 4.45 ± 0.18 Å for HPV31 (Fig. 1g). These predicted structural changes appeared to be attributable to the insertion of additional amino acid residues into the BC loops of HPV31 (Pro^58^), HPV33 (Ala^58^), HPV52 (Ser^57^, Gly^58^ and Gly^60^) and HPV58 (Asn^58^) in comparison to HPV16. No structural changes in the DE loop were predicted between the L1 homology models representing the non-vaccine Alpha-9 genotypes compared to HPV16 (Fig. 1c). The DE encircles the lumen of the capsomer (Fig. 1a) and it is the only L1 loop which does not contain any insertions or deletions within the amino acid sequences of the non-vaccine genotypes relative to HPV16. All the non-vaccine Alpha-9 homology models were predicted to have structural changes in the EF loop compared to the HPV16 model (Fig. 1d). Genotypes HPV33 and HPV58 had single amino acid residue deletions within the EF loop that correspond to Gly^183^ within the EF loop of HPV16, which may contribute towards the mean EF loop shifts of 4.11 ± 0.43 Å for HPV33 and 1.17 ± 0.14 Å for HPV58 compared to HPV16 (Fig. 1g). In contrast, the predicted structural changes in the EF loops of HPV31, HPV35 and HPV52 were not underpinned by amino acid residue insertions or deletions in comparison with HPV16.

Structural changes in the FG loop were predicted for HPV35 and HPV52 (Fig. 1e) with a mean FG loop shift compared to HPV16 of 2.65 ± 0.06 Å and 3.31 ± 0.19 Å, respectively (Fig. 1g). Both these genotypes have FG loop amino acid residue deletions (HPV35: Corresponding to Ser^280^ and Gly^281^ of HPV16) or insertions (HPV52: Asn^284^ and Ser^285^) in comparison with HPV16 which appeared to influence these predicted structural changes. The FG amino acid sequence of HPV31, HPV33 and HPV58 contained no residue deletions or insertions in comparison with HPV16, and no structural changes in the FG loop were predicted for these non-vaccine genotypes compared to HPV16. The HI loops of HPV33, HPV52 and HPV58 have an amino acid residue deletion which corresponds to Thr^350^ within the HI loop of HPV16. The three L1 homology models representing these non-vaccine genotypes were predicted to have structural changes compared to HPV16 homology model within the HI loop, in comparison to HPV31 and HPV35 for which no structural changes were predicted (Fig. 1f).

### Design and generation of chimeric PsV

The predicted structural differences between the L1 loops of the non-vaccine Alpha-9 PsV compared to the HPV16 PsV were used to inform the design of chimeric PsV with inter-genotype loop swaps. It was reasoned that the L1 loops of the non-vaccine genotypes which presented a similar topography to the corresponding L1 loop of HPV16, were more likely to be recognised by vaccine-induced cross-neutralising antibodies. Based upon this rationale, the DE loop was considered to be a candidate for further investigation based upon the predicted structural similarity between the DE loops of all the non-vaccine Alpha-9 genotypes compared to HPV16 (Fig. 1c). In contrast, the frequency of predicted structural divergence observed between the BC and EF loops (Fig. 1b,d) of the non-vaccine genotypes and HPV16 negated any further investigation of these two loops. Both the FG and HI loops (Fig. 1e,f) were chosen for further investigation based upon their predicted structural similarity between HPV16 and the non-vaccine Alpha-9 genotype HPV31 which is frequently recognised by cross-neutralising antibodies^23^,^33^.

**Figure 2:**
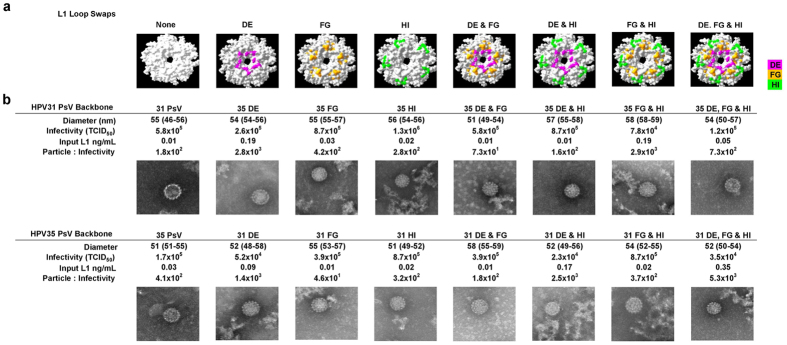
Chimeric L1L2 PsV (**a**) Top view of L1 capsomer with variable positons between HPV31 and HPV35 within the DE (pink), FG (orange) and HI (green) loops highlighted. (**b**) EM images of HPV31, HPV35 and chimeric L1L2 PsV with preparations characterised for the median (IQR) particle diameter, infectivity, L1 concentration and the resultant particle-to-infectivity ratio. TCID_50_, 50% Tissue Culture Infectious Dose.

A panel of chimeric L1L2 PsV with inter-genotype DE, FG and HI loop swaps in isolation or combination were designed and generated. The loop swaps were made between HPV31, a genotype recognised by cross-neutralising antibodies and HPV35, which was chosen as the background control since cross-neutralising antibodies demonstrate minimal recognition of this genotype. The number of amino acid residues which were substituted between HPV31 and HPV35 differed between the three loops. The DE, which is the longest L1 loop, only had five positions where the residues varied between HPV31 and HPV35. The thirty residue long FG loop had eleven variable positions whilst the HI, which is the shortest L1 loop, varied at nine of its sixteen amino acid positions. The L1L2 PsV with either a HPV31 or HPV35 backbone and single (DE, FG & HI), double (DEFG, DEHI & FGHI) or triple (DEFGHI) loop swaps (Fig. 2a) generated similarly-sized particles and particle-to-infectivity (PI) ratios. For example, the PsV with a HPV31 backbone produced a median PI ratio of 350 (IQR, 173 to 1,259) compared to a PI ratio of 392 (IQR, 284 to 1,669) produced by the PsV with a HPV35 backbone (Fig. 2b).

### Cross-neutralising antibody recognition of specific L1 domains

**Figure 3:**
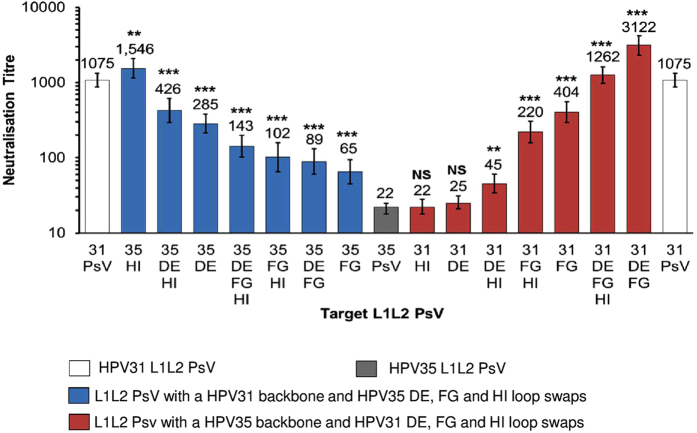
Neutralisation sensitivity of chimeric L1L2 PsV to HPV vaccine-induced antibodies. Colour indicates L1L2 PsV constructs: HPV31 (white), HPV35 (grey), HPV31 L1 backbone with HPV35 loop switches (blue) and HPV35 L1 backbone with HPV31 loop switches (red). Bar graph represents the neutralisation GMT of n = 36 HPV vaccine sera (Cervarix^®^ n = 19; Gardasil^®^ n = 17), all of which were tested against HPV31, HPV35 and the chimeric L1L2 PsV. Error bars represent GMT neutralisation titre 95% CI. *p* values obtained using the Wilcoxon paired signed-rank test represent differences in neutralisation titre between either the HPV31 PsV and the chimeric PsV with a HPV31 backbone or the HPV35 PsV and the chimeric PsV with a HPV35 backbone.

Thirty-six HPV vaccinee sera (Cervarix^®^ n = 19; Gardasil^®^ n = 17) were tested against the chimeric L1L2 PsV. The cross-neutralising titres generated against the chimeric L1L2 PsV with a HPV31 backbone were compared against the HPV31 PsV whilst the titres generated against the chimeric L1L2 PsV with a HPV35 backbone were compared against the HPV35 PsV (Fig. 3).

It was reasoned that the introduction of HPV35 loops into a HPV31 backbone would result in the reduction of cross-neutralising antibody recognition of HPV31. The incorporation of the HPV35 HI loop alone did not result in a reduction in cross-neutralising antibody recognition; however, the incorporation of the HPV35 DE loop (Fig. 3) reduced cross-neutralising antibody recognition of HPV31 by a median 4.0-fold (IQR, 2.0 to 6.5-fold; Wilcoxon paired signed-rank test, *p* < 0.001). The largest impact of any single or combination loop swap was observed with the HPV35 FG loop alone which resulted in a median decrease in cross-neutralising antibody recognition of 19.0-fold (IQR, 8.3 to 41.4-fold; *p* < 0.001) compared to the HPV31 PsV.

The transfer of cross-neutralising antibody recognition to HPV35 was not supported by either the introduction of the HPV31 DE or HI loop into the HPV35 backbone, both of which had no significant effect on cross-neutralising antibody recognition of HPV35 producing geometric mean titres (GMT) of 25 (95% CI, 21 to 31; *p* = 0.637) and 22 (95% CI, 18 to 28; *p* = 0.322), respectively, compared to the HPV35 PsV titre of 22 (95% CI, 18 to 25) (Fig. 3). Conversely, the introduction of the HPV31 FG loop into the HPV35 L1 backbone significantly increased cross-neutralising antibody recognition of the HPV35 PsV. This effect was most dramatic when the FG loop was in combination with the DE loop of HPV31, resulting in a median increase in cross-neutralising antibody recognition of 155.7-fold (IQR, 49.7 to 288.4-fold; *p* < 0.001) compared to the HPV35 PsV.

The cross-neutralising antibody recognition of the chimeric L1L2 PsV was independent of the HPV vaccine received. For example, the replacement of the FG loop within the HPV31 L1 backbone with that of HPV35 resulted in a median 17.8-fold (IQR, 8.2 to 39.8-fold; *p* < 0.001) and 20.1-fold (IQR, 10.8 to 40.5-fold; *p* < 0.001) decrease relative to the HPV31 PsV for Cervarix^®^ and Gardasil^®^ sera, respectively. The replacement of the DE and FG loops within the HPV35 L1 backbone with those of HPV31 significantly increased recognition of the HPV35 PsV (Cervarix^®^ GMT 22; 95% CI, 18 to 25; Gardasil^®^ GMT 21; 95% CI, 16 to 26) by both Cervarix^®^ (GMT 4,603; 95% CI, 3,349 to 6,325; *p* < 0.001) and Gardasil^®^ sera (GMT 2,024; 95% CI, 1,255 to 3,264; *p* < 0.001).

### Fine-mapping of cross-neutralising antibody epitope footprint

**Figure 4:**
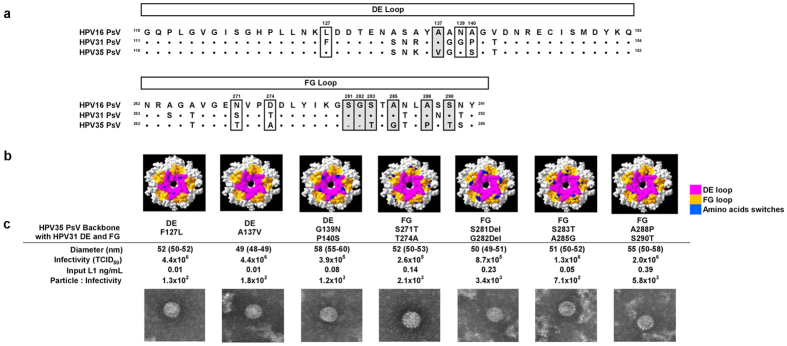
Chimeric L1L2 PsV with DE or FG loop amino acid switches (**a**) Amino acid alignment of HPV16, HPV31 and HPV35 DE and FG loops. Amino acid positions within the DE and FG loops selected for site-directed mutagenesis are highlighted with identical residues between HPV16 and HPV31 but different from HPV35 boxed in grey. (**b**) Top view of L1 capsomer with the DE (pink) and FG (orange) loops highlighted alongside amino acid positions identified for site-directed mutagenesis (blue). (**c**) EM images of chimeric L1L2 PsV preparations with a HPV35 backbone and HPV31 DE and FG loops with amino acid switches characterised for the median (IQR) particle diameter, infectivity, L1 concentration and the resultant particle-to-infectivity ratio.

In order to predict the residues within the DE and FG loops which may be involved in the epitope footprint(s) recognised by vaccine-induced cross-neutralising antibodies, the DE and FG loop amino acid sequences of HPV16, HPV31 and HPV35 were aligned (Fig. 4a). Amino acid positions for which HPV16 (vaccine type) and HPV31 (cross-neutralising antibody target) shared the same residue but HPV35 (background control) did not, were identified in the DE (Ala^137^) and FG loops (Ser^281^, Gly^282^, Ser^283^, Ala^285^, Ala^288^ and Ser^290^). These residues were selected to undergo site-directed mutagenesis to determine their contribution to the epitope footprint recognised by cross-neutralising antibodies, alongside additional sites where the residue differed between HPV31 and HPV35 (DE: Phe^127^, Gly^139^ and Pro^140^; FG: Ser^271^ and Thr^274^) (Fig. 4b).

A panel of seven chimeric L1L2 PsV with a HPV35 backbone and HPV31 DE and FG loop swaps were generated. These chimeric PsV harboured single or double amino acid residue switches incorporated in either the DE or FG loop whereby the amino acids present in HPV31 were swapped for the amino acids present at those positions in HPV35 (Fig. 4c). The PsV particles generated were of a similar size and had similar PI ratios.

**Figure 5:**
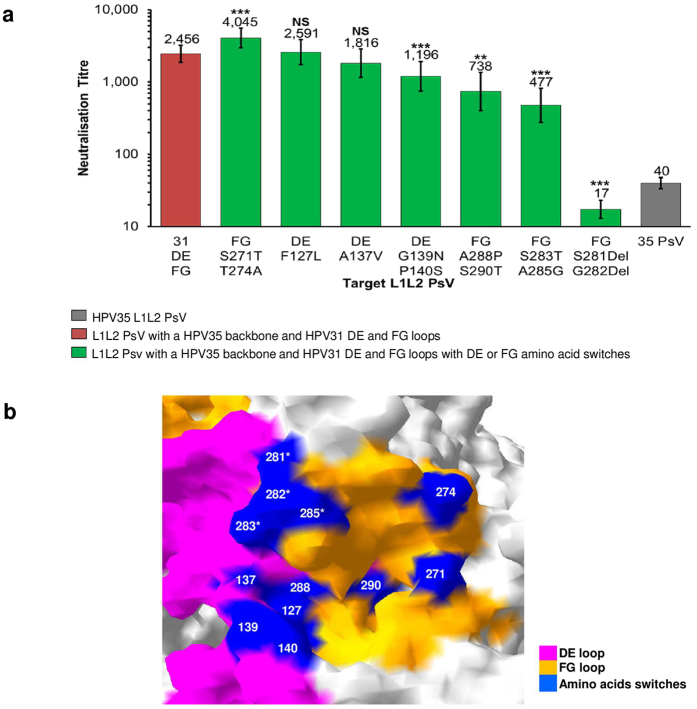
Fine-mapping of L1 epitope footprint of cross-neutralising antibodies. (**a**) Neutralisation sensitivity of chimeric L1L2 PsV with a HPV35 backbone and HPV31 DE and FG loops with amino acid switches to HPV vaccine-induced antibodies. Colour indicates L1L2 PsV constructs: HPV35 (grey), HPV35 backbone and HPV31 DE and FG loops (red), HPV35 L1 backbone with HPV31 DE and FG loop switches with DE or FG amino acids switches (green). Bar graph represents the neutralisation GMT of n = 24 HPV vaccine sera (Cervarix^®^ n = 12; Gardasil^®^ n = 12), all of which were tested against HPV35 and the chimeric L1L2 PsV. Error bars represent GMT neutralisation titre 95% CI. *p* values obtained using the Wilcoxon paired signed-rank test represent differences in neutralisation titre between the chimeric PsV with a HPV35 backbone and HPV31 DE and FG loops and the chimeric PsV with a HPV35 backbone and HPV31 DE and FG loops with DE or FG amino acid switches. (**b**) Close up view on HPV31 L1 homology model of residues identified for site-directed mutagenesis (blue) within the DE (pink) and FG (orange) loops. The mutation of positions indicated with an asterisk resulted in a ≥4-fold drop in cross-neutralisation titre compared to the chimeric PsV with a HPV35 backbone and HPV31 DE and FG loops.

A subset of twenty-four HPV vaccinee sera (Cervarix^®^ n = 12; Gardasil^®^ n = 12), from the original panel of thirty-six, were tested against the seven new chimeric L1L2 PsV and the cross-neutralising titres generated were compared against the chimeric L1L2 PsV with a HPV35 backbone and HPV31 DE and FG loops (Fig. 5a). Within the DE loop of HPV31, amino acid switches F127L and A137V did not result in a significant reduction in cross-neutralising antibody recognition; however, the dual amino switches of G139N and P140S reduced cross-neutralising antibody recognition by a median 1.6-fold (IQR, 1.2 to 2.0-fold; Wilcoxon paired signed-rank test, *p* < 0.001).

The amino acid switches within the FG were separated into the early region (HPV16 numbering: Ala^264^ to Lys^278^) and the late region (Gly^279^ to Ser^288^) of the loop. The dual amino acid switches within the late region (S283T and A285G; A288P and S290T) resulted in a significant reduction in cross-neutralising antibody recognition; however, the most dramatic impact was observed by the dual amino acid deletion at positions S281Del and G282Del which produced a cross-neutralising titre [GMT of 17 (95% CI, 13 to 27; *p* < 0.001)] lower than the titre observed against the HPV35 PsV [40 (95% CI, 33 to 47)] (Fig. 5a). In contrast, the dual amino acid switch at positions S271T and T274A within the early region did not result in a reduction in cross-neutralising antibody recognition.

Mapping of the mutagenised residues within the DE and FG loops on to the HPV31 L1 homology model demonstrated that these positions are all surface-exposed on the capsid (Fig. 5b) and that the amino acid positions within the late region of the FG which had the biggest impact upon cross-neutralising antibody recognition (S281Del and G282Del; S283T and A285G) are clustered together at the intersection between the FG and DE loops.

## Discussion

This study attempted to delineate the L1 domains recognised by HPV vaccine-induced Alpha-9 cross-neutralising antibodies. We designed and generated chimeric L1L2 PsV with inter-genotype loop swaps which were tested for their sensitivity to cross-neutralisation by antibodies elicited by HPV vaccines.

The cross-neutralising antibodies detected following immunisation with HPV16 VLP recognise L1L2 PsV representing a number of non-vaccine Alpha-9 genotypes^22^,^27^,^33^. It is reasonable to assume that the L1 proteins of these non-vaccine genotypes harbour antigenic domains which present a similar topography to HPV16. In order to identify these domains, crystallographic homology models representing the L1 amino acid sequences of the Alpha-9 PsV were generated and utilised for the comparison of loop structures. Predicted structural differences between HPV16 and the non-vaccine Alpha-9 genotypes were common in the BC and EF loops located on the outer rim of the L1 capsomer but occurred less frequently in the FG and HI loops, and no structural differences were predicted for the centrally-positioned DE loop.

The surface-exposed L1 loop regions of the HPV capsid demonstrate a higher degree of sequence heterogeneity than the core regions as evident from the higher levels of intra- and inter-genotype amino acid variability located to the loops^11^,^32^. It is thought that this diversity is driven by the pressure to escape neutralising antibodies which target epitopes within the surface exposed loops^8^. Amino acid residues within the L1 loops also appear to play critical roles in maintaining capsid stability^36^ and initiating virus-host interactions^9^ which may account for the structural similarity predicted between HPV16 and the non-vaccine Alpha-9 genotypes in some L1 loops. The computer modelling of L1-L2 protein interactions suggests that regions of the DE and FG loops interact with proline-rich regions of the L2^36^ whilst a cysteine residue within the EF loop is crucial for the formation of inter-capsomeric L1-L1 disulphide bonds^6^,^37^. Additionally, the BC, FG and HI loops contain lysine residues which can facilitate binding to heparin sulfate proteoglycans, the initial step required for successful HPV infection^9^.

The analysis of L1 loop structure within this study has to be interpreted with the caveat that they are structural predictions derived from homology models representing the Alpha-9 PsV L1 amino acid sequence rather than the resolved L1 crystal structures. Nevertheless homology modelling is a standard approach used for H1N1 pandemic influenza^38^, HBV^39^ and HIV^40^ in order to predict the location of antigenic domains and sites of protein-protein interactions. The pairwise comparison between the HPV35 L1 homology model based upon the HPV16 capsomer crystal structure and the HPV35 L1 capsomer crystal structure^41^, demonstrated a close structural relationship. These data imply that in the absence of available L1 crystal structures, homology models have utility as surrogate structures for the prediction of structural differences between L1 proteins.

The L1 loops of HPV16 have been investigated extensively using L1 VLP as target antigens for antibody recognition. The manipulation of individual L1 loops by the insertion of foreign B-cell epitopes from HIV and HBV identified the FG and HI loops as immunogenic regions of the L1 capsid^42^,^43^,^44^. Loops with amino acid point mutations and VLP with complete inter-genotype loop switches have identified residues which contribute to the epitope footprints recognised by type-specific neutralising murine MAbs^10^,^45^,^46^. The use of functional chimeric L1L2 PsV which measure antibody specificities capable of neutralising PsV infectivity has, however, been limited to a single HPV16 construct with a HPV33 BC loop swap used to map the epitope of a HPV33 L1 MAb (H33.J3)^45^.

The limited use of chimeric L1L2 PsV as antigenic targets may reflect the reduced tolerance of these complex particles to manipulation. Within this present study, the chimeric L1L2 PsV consisted of inter-genotype loop swaps between two genotypes from within the same species group (Alpha-9: HPV31 and HPV35) an approach which resulted in the successful formation of functional chimeric L1L2 PsV particles. Chimeric PsV have been utilised as antigens in other virus systems including HIV^47^, H5N1 avian influenza^48^ and JC polyomavirus^49^ to map polyclonal antibody recognition of specific antigenic domains.

The chimeric L1L2 PsV demonstrated that the FG loop is necessary and sufficient for the epitope footprints of a significant proportion of cross-neutralising antibody specificities and that the DE loop appears to enhance this recognition. The HI loop appeared not to contribute towards cross-neutralising antibody recognition. Two or more L1 loops appear to contribute towards the majority of identified L1 MAb epitopes, although there are instances where a single loop has supported an epitope footprint, for example the BC loop alone is targeted by some HPV6, HPV33 and HPV16 L1 MAbs^45^,^50^,^51^. The epitope footprints of four neutralising HPV16 MAbs have recently been resolved using cryo-electron microscope analysis and demonstrated for the first time that, alongside FG and HI loop residues, residues in the DE loop formed the core of each epitope with minor contributions from residues in the BC and EF loops^14^,^15^. Amino acid residues within the DE loop also formed the majority of the epitope footprint recognised by the HPV16 neutralising human MAb 26D1, which was isolated from a HPV vaccine recipient^52^. In this present study, we found that the DE loop of HPV31 enhanced cross-neutralising antibody recognition of the HPV31 FG loop within the HPV35 L1 backbone. This enhancement may result from DE loop residues functioning as part of the epitope footprint or maybe due to DE loop interactions which support the optimum presentation of the FG loop.

Fine-mapping of the epitope footprint recognised by cross-neutralising antibodies was undertaken, whereby residues at DE and FG loop amino acid positions of HPV16 and HPV31 which differed from HPV35 were subjected to site-directed mutagenesis alongside sites where the residues were variable between all three genotypes. Cross-neutralising antibodies appeared to primarily target amino acid residues located in the late region of the FG loop particularly the amino acids at positions 281 and 282, the deletion of which resulted in almost complete loss of recognition by cross-neutralising antibodies. In contrast, the mutation of two amino acid positions in the early region of the FG loop had no effect upon cross-neutralising antibody recognition, indicating that the early region is not a target for such antibody specificities.

The FG loop contains a Lys^278^ (HPV16 numbering) which mediates primary host attachment^9^, an interaction which is inhibited *in vivo* by vaccine induced L1 antibodies^53^ providing a possible mechanistic reason behind the antigenic targeting of the FG loop by both type-specific MAbs and natural infection antibodies^13^,^16^,^45^,^51^,^54^. The early region of the FG loop is known to harbour residues involved in the epitope footprints of type-specific, neutralising HPV31 MAbs^16^ whilst the late region contains the majority of residues involved in the epitope footprints of type-specific, neutralising HPV16 MAbs^14^. The data in this study appear to demonstrate that the late region of the FG loop harbours the amino acid residues recognised by HPV vaccine-induced cross-neutralising antibodies and that direct overlap exists with residues in the HPV16 type-specific antibody footprints. This observation suggests that HPV31 type-specific antibodies and HPV31 cross-neutralising antibodies generated against HPV16 recognise different L1 domains and that the domain recognised by HPV31 cross-neutralising antibodies is subdominant within the epitope footprint of HPV16 type-specific antibodies.

Cross-neutralising antibodies appear to recognise a single non-vaccine Alpha-9 genotype or multiple non-vaccine genotypes, in addition to HPV16^55^. It is likely that these antibody specificities target distinct and overlapping epitope footprints which share a common domain with HPV16. The generation of L1 MAbs which can cross-neutralise non-vaccine Alpha-9 genotypes may further our understanding of the L1 domains recognised by cross-neutralising vaccine antibodies and their relationship to type-specific epitopes in the context of the capsid surface.

We propose that the L1 domain recognised by inter-genotype cross-neutralising antibodies elicited by the HPV vaccines comprise residues within the late region of the FG loop possibly stabilised by one or more residues within the DE loop. The cross-neutralising antibodies which target this L1 domain may play a role in HPV vaccine-induced cross-protection.

## Materials and Methods

### Generation of L1 Homology Models

L1 homology models were created from the L1 amino acid sequences of L1L2 PsV representing HPV16, HPV31, HPV33, HPV35, HPV52 and HPV58 (sequences available from http://home.ccr.cancer.gov/LCO/packaging.htm) using SWISS MODEL (http://swissmodel.expasy.org/)^56^,^57^. The crystal structure of the HPV16 L1 capsomer (PDB code: 2R5H)^41^ was used as the template to which the target L1 amino acid sequences of the Alpha-9 PsV were modelled. The quality of a predictive model is measured by the GMQE score which ranges from 0 to 1 and represents the expected accuracy of the resulting model, with a score of 1 indicating the highest level of quality estimation reliability^58^.

### L1 Modelling

DeepView Swiss-Pdb viewer v4.0^59^ was used to perform pairwise L1 model comparisons by superimposition and predicted structural differences between models were measured in Å. The superimposition of L1 homology models was supported by a RMS value, which represents the average Å distance between corresponding atoms in the two models. The lower the RMS value the closer two models are related, with a model compared to itself generating an RMS value of 0^59^. The crystal structure of the HPV35 L1 capsomer (PDB code: 2R5J)^41^ was used as a pairwise comparison control for the HPV35 L1 homology model. The DeepView programme was additionally used to model the positions of amino acid residues of interest on to the L1 homology models.

### L1L2 PsV

The L1 genes with HPV31 or HPV35 backbones and reciprocal inter-genotype loop swaps were synthesised by GeneArt^®^ (Thermo Fisher Scientific) and were applicable site-directed mutagenesis was carried out using the QuikChange kit (Stratagene). L1 genes were subcloned into the p31sheLL or p35sheLL plasmids in conjunction with the Rapid DNA Dephos & Ligation kit (Roche). HPV31, HPV35 and chimeric L1L2 PsV carrying a luciferase reporter were expressed and purified as previously described^33^. Particle formation and size were determined by electron microscopic analysis of negatively stained particles. Ten PsV particles were measured in nanometres (nm) from each preparation and the median diameter and inter-quartile range calculated (Fig. 2b). The equivalent of a 50% Tissue Culture Infectious Dose (TCID_50_) was estimated for each PsV preparation using the Spearman-Karber equations and a standardised input of 300 TCID_50_ was used for all PsV^33^. The L1 concentrations of PsV stocks were estimated by semiquantitative L1 Western blot analysis using CamVir-1 antibody (Abcam, United Kingdom). Particle-to-infectivity ratios were determined on the basis of an estimated particle amount of 3 × 10^7^ particles per ng L1 protein (http://home.ccr.cancer.gov/lco/production.asp), with the ratio being normalized for the input volume and the TCID_50_.

### Study Samples

Serum samples were available from 12–15 year old girls randomised to receive three doses of Cervarix^®^ or Gardasil^®^ as part of a Phase IV clinical trial comparing HPV vaccine immunogenicity (www.clinicaltrials.gov: NCT00956553; REC number 09/H0720/25). A subset of samples (Cervarix^®^ n = 19; Gardasil^®^ n = 17) taken 1 month after receiving the final dose, were selected for analysis based upon HPV31 cross-neutralisation antibody titres^23^.

### L1L2 PsV Neutralisation Assay

Samples were subjected to 5 serial dilutions, with the antibody titre resulting in an 80% reduction in the luciferase signal produced by control wells containing PsV and cells only estimated by interpolation. HPV antibody control reagents were included in each assay run^60^ alongside heparin (H-4784; Sigma-Aldrich) which was used as a positive inhibitor control: Median heparin concentration (μg/mL) against PsV HPV31 5.5 (IQR, 3.8 to 6.7; n = 6) and HPV35 3.1 (IQR, 2.5 to 3.3; n = 6); Median neutralisation titres of the positive antibody control reagent (high titre HPV16/18) against PsV HPV31 389 (IQR, 333 to 427; n = 12) and HPV35 46 (IQR, 42 to 53; n = 12). The negative antibody control reagent (HPV negative) had a titre of <40 in all assays (n = 24).

### Statistical analysis

The Wilcoxon paired signed-rank test was used for the comparison of cross-neutralising antibody titres between different L1L2 PsV targets. The test was performed using the statistical package, Stata 12.1 (StataCorp LP).

## Additional Information

**How to cite this article**: Bissett, S. L. *et al*. The DE and FG loops of the HPV major capsid protein contribute to the epitopes of vaccine-induced cross-neutralising antibodies. *Sci. Rep.* **6**, 39730; doi: 10.1038/srep39730 (2016).

**Publisher's note:** Springer Nature remains neutral with regard to jurisdictional claims in published maps and institutional affiliations.
